# Integrating mathematical models with experimental data to investigate the within-host dynamics of bacterial infections

**DOI:** 10.1093/femspd/ftaa001

**Published:** 2020-01-14

**Authors:** Myrto Vlazaki, John Huber, Olivier Restif

**Affiliations:** Department of Veterinary Medicine, University of Cambridge, Madingley Road, CB3 0ES, Cambridge, UK

**Keywords:** mathematical biology, within-host dynamics, host–pathogen interactions, mechanistic model, parameter inference

## Abstract

Bacterial infections still constitute a major cause of mortality and morbidity worldwide. The unavailability of therapeutics, antimicrobial resistance and the chronicity of infections due to incomplete clearance contribute to this phenomenon. Despite the progress in antimicrobial and vaccine development, knowledge about the effect that therapeutics have on the host–bacteria interactions remains incomplete. Insights into the characteristics of bacterial colonization and migration between tissues and the relationship between replication and host- or therapeutically induced killing can enable efficient design of treatment approaches. Recently, innovative experimental techniques have generated data enabling the qualitative characterization of aspects of bacterial dynamics. Here, we argue that mathematical modeling as an adjunct to experimental data can enrich the biological insight that these data provide. However, due to limited interdisciplinary training, efforts to combine the two remain limited. To promote this dialogue, we provide a categorization of modeling approaches highlighting their relationship to data generated by a range of experimental techniques in the area of *in vivo* bacterial dynamics. We outline common biological themes explored using mathematical models with case studies across all pathogen classes. Finally, this review advocates multidisciplinary integration to improve our mechanistic understanding of bacterial infections and guide the use of existing or new therapies.

## Teaser

This review illustrates the added value of integrating mathematical models with experimental data to investigate the within-host dynamics of bacterial infections.

## INTRODUCTION

Bacterial infections have historically been classified amongst the leading causes of death in humans (World Health Organisation 2019). The discovery and clinical introduction of antibiotics during the 20th century significantly reduced the morbidity and mortality of bacterial diseases (Yoshikawa 2002). However, the rise of resistance to most first-line antibiotics (reviewed by Hofer 2018) and antibiotic tolerance (reviewed by Brauner *et al*. 2016) compounded by inequities in antimicrobial access (Center for Disease Dynamics, Economics and Policy 2019) persistently undermine efforts to lower the burden of bacterial diseases.

In light of these challenges, immunization of vulnerable populations (Breiman *et al*. 2012) and pathogen-specific optimization of antibiotic regimens (Meylan, Andrews and Collins 2018) have emerged as promising management strategies. However, neither novel antibiotic agents (Silver 2011) nor new vaccines are being developed fast enough (Pronker *et al*. 2013) to keep bacterial diseases in check. Even for most vaccines currently in use the mode of action at the level of host–pathogen interactions remains obscure (Oyston and Robinson 2012), while lack of an integrated understanding of the dysfunctional host–pathogen interactions targeted by antimicrobial agents leads to the underutilization of their therapeutic potential (Munguia and Nizet 2017). Concomitant with efforts to boost the rates of discovery of novel therapeutics, it is imperative to optimize the use of already available agents by disentangling the complex interactions between host immunity and pathogen behavior (Gjini and Brito 2016) and identifying the determinants of successful disease establishment and progression (Casadevall and Pirofski 2001; Munguia and Nizet 2017).

From first principles, therapeutic interventions should aim to reduce or eliminate the pathogenic bacteria from the infected host. This outcome can be achieved by slowing down bacterial replication, accelerating bacterial killing, altering bacterial migration between tissues or any favorable combination thereof. Although bacterial growth and dissemination have been extensively studied in terms of molecular and cellular mechanisms (reviewed by Ribet and Cossart 2015; Endesfelder 2019), the efforts to quantify these processes and their change in response to therapeutic interventions remain limited (Levin and Antia 2001). Quantification of these dynamics requires a detailed observation of within-host bacterial behavior by means of suitable experimental studies and high-resolution imaging and tracking technologies. However, despite the rapidly evolving technological advancements that have improved the resolution of experimental observations, many biological phenomena of interest still remain directly unobservable. This observational gap can be partly filled through quantitative inferences about the missing information with the use of mathematical models applied to relatively coarse experimental data.

In this review, we categorize mathematical models according to their appropriateness for capturing different characteristics of infection progression, and their degree of dependence on experimental data. We focus on mechanistic, data-driven models and explain their contribution to applied microbiological research. We outline commonly used experimental techniques in the study of within-host bacterial dynamics and discuss ways in which their output has been or can be enriched by suitable mathematical approaches. Finally, we broaden our scope to models applied to viral and parasitic infections to showcase a number of biological themes that have been successfully addressed using such modeling techniques.

## EXPERIMENTAL AND MATHEMATICAL MODELS AS REPRESENTATIONS OF BIOLOGICAL SYSTEMS

In a biological context, a model is defined as a simplified representation of a system or phenomenon summarizing knowledge of that system in a usable form (Eykhoff 1974). While experimental models are physical representations of the real-life system either *in vivo* or *in vitro*, mathematical models are conceptual and usually formulated as systems of mathematical equations processed either analytically or numerically (Motta and Pappalardo 2013).

Though microbiologists are familiar with experimental models, they are less so with the versatility of mathematical modeling approaches, largely due to the limited interdisciplinary training in biological sciences (Levin and Antia 2001). In this section, we aim to provide a convenient introduction to mathematical models by classifying modeling techniques according to their comparative strengths and applications to microbiological questions.

**Figure 1.**
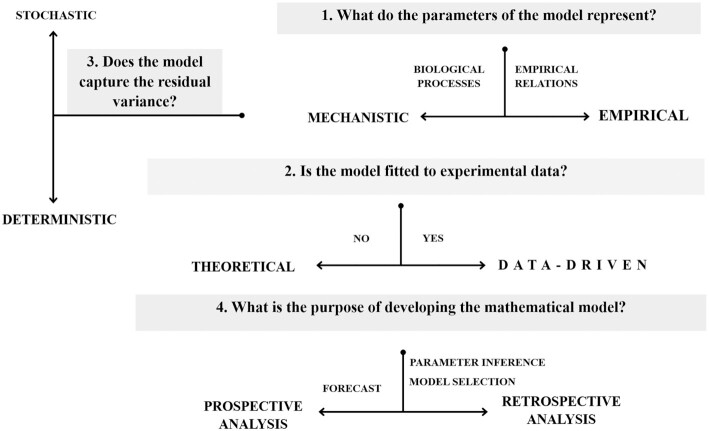
Schematic representation of the relationships between different mathematical modeling techniques. Mathematical models can be divided into mechanistic and empirical on account of whether their parameters represent biological processes or simply characterize relations between variables in the data. While empirical models are necessarily data-dependent, mechanistic models can either be system-specific and fitted to data or, generic, explorative and not related to experimental data (theoretical). Additionally, depending on the biological question addressed, mechanistic models can be deterministic when only the average behavior of the system is of interest, or stochastic when unexplained variation in the behavior of the system matters too. Mechanistic data-driven models can serve different purposes: they can either be solved forward in time to make a forecast (prospective analysis) or can be solved backwards in time to perform parameter inference and model selection (retrospective analysis).

Mathematical models can be categorized into functional classes (Fig. 1), according to different criteria such as (i) whether their parameters represent biological processes, (ii) whether they are fitted to experimental data, (iii) whether molecular, cellular or other sources of variability affect the output of the model and (iv) what the purpose of model development is.

### Mechanistic and empirical models

Mathematical models can be classified as empirical or mechanistic. Empirical models describe relations between the variables in an experimental data set, without addressing what biological mechanisms may intrinsically drive the observed patterns in the data. These relations can be quantified by parameters, whose values can be estimated using statistical analysis. Empirical models are also known as extrinsic because they do not incorporate any knowledge or hypotheses about the inner structural connectivity of the system; rather they are only based on its observable, external behavior (Thakur 1991).

**Figure 2.**
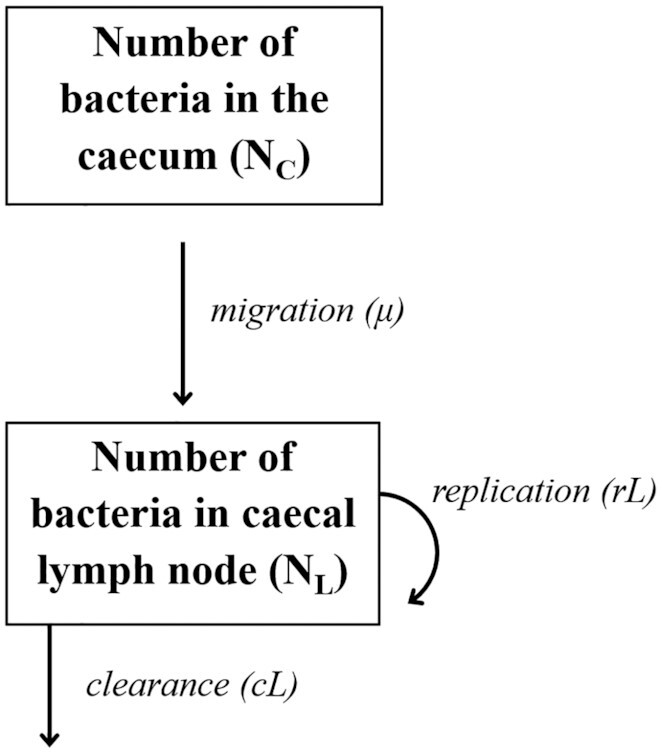
An example of a flow diagram as a schematic representation of mechanistic models in microbiology (adapted from Kaiser *et al*. 2013). Boxes represent the variables of the system, in this case the number of bacteria in the caecum and caecal lymph node respectively. Mathematically these variables are shortened for convenience as NC and NL. The processes that change the state of the system are represented by arrows and, here, correspond to bacterial migration, replication and clearance. The rates at which these processes take place are quantified by parameters, in this case, μL, rL and cL corresponding to the rates of migration, replication and clearance, respectively.

By contrast, mechanistic models incorporate the biological mechanisms by which changes in the system are thought to occur and require some knowledge or speculation about the unobserved interactions that determine the observable output (Thakur 1991; Baker *et al*. 2018). In the context of mathematical modeling, a mechanism often does not correspond to the molecular, cellular or genetic hierarchy that collectively constitutes causality from a microbiologist's viewpoint. From a modeler's point of view, a mechanism is a conceptual representation of the process acting to change the state of the system in a certain direction. Structurally, mechanistic models can be represented with flow diagrams, whereby the state of the biological system, i.e. an imaginary complete snapshot of that system at a given point in time, changes through processes measured by parameters in the model (Fig. 2). Examples of biologically relevant quantities encoded as variables in these models (i.e. boxes in the flow diagram) can include concentrations of cytokines and antibodies, as well as numbers of immune cells and infectious agents in tissues amongst others. These quantities are at times directly measurable experimentally and at others only determinable by proxy.

### Data-driven and theoretical models

The second relevant classification of mathematical models is that into data-driven and theoretical. While empirical models are, by definition, data-driven, mechanistic models can be of either class. Mechanistic data-driven models are usually system-specific and parameterized based on experimental outputs. For example, in 2014, Coward *et al*. carried out an experiment using individually tagged strains of *Salmonella* Typhimurium to determine the effects of different vaccines on the rates of replication and killing of bacteria. The measurements of bacterial numbers in the differentially tagged subpopulations along the infection timeline were fed into a population-based mathematical model, which permitted estimation of the rates of replication and killing of bacteria under the two immunization regimens enabling the direct comparison between them.

On the other hand, theoretical models constitute a spectrum depending on the degree to which their parameterization is empirically informed. At one end of this spectrum, there are purely theoretical models, which may describe a general pattern of infection without reference to a particular host–pathogen interaction. For example, Antia, Levin and May (1994) developed a general, theoretical model to investigate the relationship between the host's immune system and the virulence of a generic microparasite. They found that pathogens with intermediate replication rates tend to dominate their host and achieve the highest inter-host transmissibility.

Further along the spectrum, there are theoretical models referring to a specific host–pathogen system but arbitrarily parameterized with biologically plausible values. Cooper and Julius (2011) explored a theoretical model of bacterial persistence with short- and long-term dormancy and used a simulation-based approach, whereby some parameters were allowed to vary across a biologically plausible range, to conclude that the infinite-time-horizon optimal treatment strategy is not unique.

Finally, at the other end of the spectrum, there are empirically informed theoretical models, which use parameter values from a range of studies, with the potential caveat that their variable experimental sources, initial conditions or even host species may be incongruent. This limitation is counterbalanced by the benefit of maximizing information through data integration across studies and scales. For example, a substantial body of modeling work on the within-host dynamics of *Mycobacterium tuberculosis* has used diverse experimental data sets focusing on different aspects of the immune response elicited in the lungs of human, murine and simian hosts (reviewed by Kirschner *et al*. 2017). Such models have attempted to integrate data at different scales (from molecular, cellular to organ- and organism-level) and simulate the response to different vaccines and antibiotic regimes. Pienaar *et al*. (2015, 2016) used a theoretical model partly calibrated on data derived from non-human primates and rabbits to predict the efficacy of rifampin and isoniazid combination treatment regimens and to show that bacteria residing within macrophages constitute a reservoir for the development of resistance against first-line anti-TB antibiotics.

### Deterministic and stochastic models

Mechanistic models can be further subdivided into deterministic and stochastic. Deterministic models follow a predetermined trajectory given a set of starting conditions and rates at which processes evolve in time. That is, a given parameter set will always yield the same model output. By contrast, stochastic models yield different results each time when initialized with the same parameters and initial conditions. While multiple realizations of stochastic models recapitulate the range of potential outcomes and their likelihood of occurrence, the output of a deterministic model corresponds to the mean outcome of these realizations. Stochastic models can quickly become too complex to solve analytically, and their exploration may, thus, be entirely simulation-based. When stochastic processes are simulated, the random nature of trajectories generated are due to randomly, exponentially distributed, picked times until the next process, with a randomly picked process executed during that time (Gillespie 1977). This can become computationally expensive.

Despite the higher computational cost of stochastic models, they often constitute the only reasonable modeling choice, when the behavior of the system in question is particularly influenced by stochasticity. With terminology borrowed from the field of ecology, the uncertainty in the outcome can be decomposed as a function of two sources of stochasticity: demographic (e.g. Shaffer 1981; Burgman, Ferson and Akcakaya 1993) and environmental. Demographic events include births, deaths and migration of individuals. The rate at which a demographic event occurs is defined as the inverse of the average time it takes for the event to take place and can be quantified with mathematical models. These events are described by binary random variables with a certain probability of occurring per given unit of time. As demographic events at the population-level are a function of the sum of demographic events at the individual level, the strength of demographic stochasticity is greater for small populations (Kokko and Ebenhard 1996).

On the other hand, environmental stochasticity is independent of the individual; rather, it refers to unpredictable changes in the environmental conditions that the individuals experience. As such, its effects do not depend on the population size, but on the number, heterogeneity and stability of factors influencing individual behaviour (Fujiwara and Takada 2017). In the context of within-host infectious dynamics, the nature of the pathogen determines the strength of each source of stochasticity. For infections requiring a small founding population, demographic stochasticity becomes important as they may either be successful or become extinct. At the same time, the complexity of the immune response, the microenvironment of the tissue(s) colonized, and the heterogeneity of infectious foci determines the effect of environmental stochasticity on the population. When prior biological knowledge indicates that either source of stochasticity may be strong, it is advisable to use stochastic rather than deterministic models.

HIV is a good case in point to illustrate how considerable these effects can be. The first models of within-host HIV dynamics were deterministic (Nowak and Bangham 1996), but failed to account for extinction events. Later biological insights about the low probability of HIV transmission per coital act gave rise to the hypothesis that upon low level viral transmission, extinction of the infection may be more probable than take-off. Stochastic HIV models were then introduced to test this hypothesis (Pearson, Krapivsky and Perelson 2011) and have now been established as an important tool in the HIV modeling literature.

### Prospective and retrospective analysis

Mathematical models can be used for prospective or retrospective analysis, according to their intended purpose in a study. Models can have a forward solution, when the initial conditions and parameters are known or chosen *a priori* by the modeller. They can predict what the state of the system will be at different timepoints in the future under different conditions. One common application of prospective modeling is the comparison of the effect of therapeutic interventions on infectious load reduction (e.g. Grant *et al*. 2008; Pienaar *et al*. 2015).

A model can also be solved backwards when the parameters are unknown but the outputs at different time-points are known. When analyzed retrospectively, mechanistic models can be used to infer the unknown parameters. The inference process explores the parameter space, which consists of all allowed parameter combinations. For each parameter combination, the model is used to predict the state of the system at time points on interest. The state of the system, as predicted by the model given a set of parameters, is then compared to the experimental observations using statistical tools. The parameter set whose corresponding predicted output is the closest to the experimentally observed data is taken as the best parameter estimate resulting from the inference process. An example of a mechanistic model analyzed retrospectively with the purpose of parameter inference is provided by Dybowski *et al*. (2015). They infected mice with a mixture of isogenically tagged bacteria derived either from liquid culture or recovered from previously infected mice. They fitted a mechanistic model to experimental measurements of numbers of bacteria per tagged strain to infer the unobserved replication and killing rates of bacteria and concluded that *in vivo* passage of bacteria affects their within-host dynamics in subsequent infections.

Mechanistic models, analysed retrospectively, can also be used in the context of model selection to address competing hypotheses about a biological process and these hypotheses can be tested by fitting the models to experimental data. Models with poor fit are unlikely to represent plausible candidates for the underlying biological mechanism. For instance, Handel, Longini and Antia (2009) tested different hypotheses about the immune response to influenza A. Using model selection, they rejected the hypothesis that regrowth of epithelial cells affects the rate at which the infection progresses and highlighted the need for additional experimental data to test more detailed hypotheses about this immune response.

It is important to note that the prospective and retrospective features of models are not mutually exclusive. A model can be used retrospectively and prospectively for both parameter inference and forecast, respectively. Parameters can be inferred by solving the model backwards using a fraction of the observed measurements. Then, the model, parameterized with the estimated values, can be used to predict future outcomes (forward solution). If the predicted outcomes match the remaining experimental observations, the model can be validated (Steyerberg and Harrel Jr 2016).

## MATHEMATICAL MODELS INTEGRATED WITH EXPERIMENTAL TECHNIQUES

Previously, we used examples to illustrate how different forms of mathematical modeling can be used to understand different aspects of host–pathogen interactions. The added value that a data-based mathematical model brings depends on multiple factors, including the understanding of the biological processes driving the system, the fidelity with which the putative biological knowledge has been translated to a mathematical model, and the quality and resolution of the experimental data used in model calibration. While the former two factors depend on the knowledge about the biological system of interest, the latter depends on the availability of relevant technology and know–how, and on a good understanding of the format and quality of experimental output required by the model.

This section briefly reviews experimental techniques commonly used to observe bacterial growth *in vivo* and outlines the scope for their use with mathematical models. Table 1 summarizes the studies that have hitherto attempted to characterize processes that shape the within-host bacterial dynamics, highlighting the nature of the experimental technique used, whether the setup characterized the process at the level of the single bacterium or the entire population, and whether an average or a distribution of the unit (e.g. rate or elapsed generations) was obtained. This structured framework of pairwise technique-model combinations aspires to serve as a preliminary template for lab-based microbiologists to consider ways in which their experimental outputs could be made amenable to mathematical analysis.

**Table 1.** Summary of studies characterizing processes shaping bacterial dynamics

Marker	Detection Technique	Characterized Process	Population/Individual	Average/Distribution	Species	Reference
Plasmid segregation	Plating and enumeration	Replication, death	Population	Average	*S. enterica* *	Hormaeche (1980)
					*S. enterica* *	Maw and Meynell (1968)
					*E. coli* *	Meynell (1959)
					*S. enterica* *	Gulig and Doyle (1993)
					*H. influenzae* *	Moxon and Murphy (1978)
					*L. monocytogenes* *	Bakardjiev, Theriot and Portnoy (2006)
					*S. aureus* *	McVicker *et al*. (2014)
					*E. coli* **	Frenoy and Bonhoeffer (2018)
Signature-tagged strains	qPCR	Replication, death, migration, colonization	Population	Average	*S. enterica* *	Grant *et al*. (2008)
	qPCR				*L. monocytogenes* *	Melton-Witt *et al*. (2011)
	qPCR				*E.coli* *	Schwartz *et al*. (2011)
	qPCR				*E. coli* *	Walters *et al*. (2012)
	qPCR				*S. enterica* *	Kaiser *et al*. (2013)
	qPCR				*B. anthracis* *	Lowe *et al*. (2013)
	qPCR				*S. enterica* *	Coward *et al*. (2014)
	Sequencing				*S. pneumoniae* *	Gerlini *et al*. (2014)
	qPCR				*S. enterica* *	Kaiser *et al*. (2014)
	qPCR, Sequencing				*S. enterica* *	Lim *et al*. (2014)
	qPCR				*S. enterica* *	Lam and Monack (2014)
	qPCR				*S. enterica* *	Maier *et al*. (2014)
	PCR				*B. burgdorferi* *	Rego *et al*. (2014)
	Sequencing				*V. cholerae* *	Abel *et al*. (2015)
	qPCR				*S. enterica* *	Dybowski *et al*. (2015)
	SB				*Y. pestis* *	Gonzalez *et al*. (2015)
	Sequencing				*S. enterica* *	Rossi *et al*. (2017)
	Sequencing				*L. monocytogenes* *	Zhang *et al*. (2017)
	Sequencing				*M. tuberculosis* *	Martin *et al*. (2017)
	FACS		Individual	Distribution	*E.coli* ***	Roostalu *et al*. (2008)
Fluorescent marker	FACS	Replication			*S. enterica* *	Helaine *et al*. (2010)
	FACS				*S. enterica* *	Claudi *et al*. (2014)
	FC				*S. enterica* *	Helaine, Cheverton and Watson (2014)
	FC		Population	Average	*E.coli* ***	Myhrvold *et al*. (2015)
		Colonization				
Multiple fluorescent strains	FM				*S. enterica* *	Sheppard *et al*. (2003)
	FM				*S. enterica* *	Brown *et al*. (2006)
	CLSM				*E. coli* *	Schwartz *et al*. (2011)
	LI				*B. anthracis* **	Plaut *et al*. (2012)
	LI				*S. aureus* *	Prajsnar *et al*. (2012)
	FM				*V. cholerae* *	Millet *et al*. (2014)
Peak-to-trough ratio	Sequencing	Replication	Population	Average	Mixed bacteria*	Korem *et al*. (2016)

* *Ex vivo*.

** *In vivo*.

*** *In vitro*.

FACS = Fluorescence-activated cell sorting; FM = Fluorescence microscopy; SB = Southern dot blot analysis; qPCR = quantitative polymerase chain reaction; LI = Live imaging; FC = Flow cytometry; CLSM = Confocal laser scanning microscopy.

+ Data analyzed using mechanistic mathematical models.

### Marker-based methods

The first marker-based methods used to measure the population-averaged rate of bacterial replication *in vivo* were additive, in the sense that they involved an accessory genetic element, including superinfecting bacteriophages (Hormaeche 1980; Meynell 1959; Maw and Meynell 1968), temperature-sensitive plasmids (Gulig and Doyle 1993) or plasmids carrying antimicrobial resistance genes (Moxon and Murphy 1978). In these techniques, genetic elements that induce phenotypic changes are introduced into bacteria and upon division segregate to daughter cells, leading to a reduction in the concentration of the marker, as the bacteria divide. The growth of the bacterial population can be modeled with the generation time of the population, signifying the half-life of the marker. At each bacterial generation, we would expect to find one-half less of the bacteria in the population harboring the non-genetic marker.

Additive marker-based methods have fallen out of favor for higher-resolution experimental techniques, as their output, which is based on detection of phenotypic differences, limits the potential for mathematical inference. Because the concentration of the marker decays with each subsequent bacterial division, the technique is limited to a finite number of generations and is only appropriate for studying the early stages of infection. Additionally, the possible detectable phenotypes constitute a limiting factor for the resolution of this technique.

To overcome these limitations, non-phenotypic marker-based techniques based on modifying the bacterial genome were developed, offering the added advantages of unrestricted observational potential in time, tracking of inter-organ bacterial migration, and increased resolution thanks to a broader range of possible unique markers. In contrast to their predecessors, they introduce modifications to the core bacterial genome, in the form of uniquely identifiable nucleotide sequences inserted in non-coding regions of bacterial chromosomal DNA to generate a pool of iso-phenotypic, genetically distinguishable bacteria. This bacterial pool is then inoculated into experimental models. Animals are sacrificed at time-points of interest, organs harvested, and bacterial composition determined by quantitative polymerase chain reaction (qPCR) or sequencing. Infection with signature-tagged strains allows one to follow the course of infection in the infected animal. Because the bacteria are uniquely marked, one can essentially take snapshots of the infection and determine the rates at which bacteria replicate, migrate and die in tissues at any time during the course of infection.

With non-phenotypic marker-based techniques, hosts are inoculated with a mixture of uniquely tagged bacterial subpopulations, each of which evolves as an independent infection *in vivo*. Unlike the few unique phenotypes used as markers in the additive marker-based methods, the multiplicity of infections per model animal using this new generation of non-phenotypic techniques reduces the number of hosts required to yield a sufficient volume of data to decipher the unobserved dynamics and allow the quantification of replication, killing and migration of bacteria *in vivo* (Barnes *et al*. 2006; Grant *et al*. 2008; Melton-Witt *et al*. 2011; Coward *et al*. 2014). The recent introduction of next-generation sequencing in place of qPCR for identification and quantification of the uniquely tagged strains has led to even higher-resolution data (Zhang *et al*. 2017; Abel *et al*. 2015).

DNA barcoding experiments have served as an archetype of successfully pairing mathematical with experimental approaches. On many occasions, genetic barcodes have been used in the absence of mathematical models to provide a qualitative assessment of the within-host dynamics of bacterial infections (Zhang *et al*. 2017; Walters *et al*. 2012; Lim *et al*. 2014; Abel *et al*. 2015). In a standard infection, observation of a change from a dissimilar to a highly similar composition of tagged populations between two tissue samples would indicate bacterial migration between them, while the sudden loss of tagged strain diversity would suggest a rapid bottleneck. For instance, Walters *et al*. (2012) used isogenic tagging experiments to show a bottleneck in the uropathogenic *Escherichia coli* infection between the kidney and the bloodstream.

**Figure 3.**
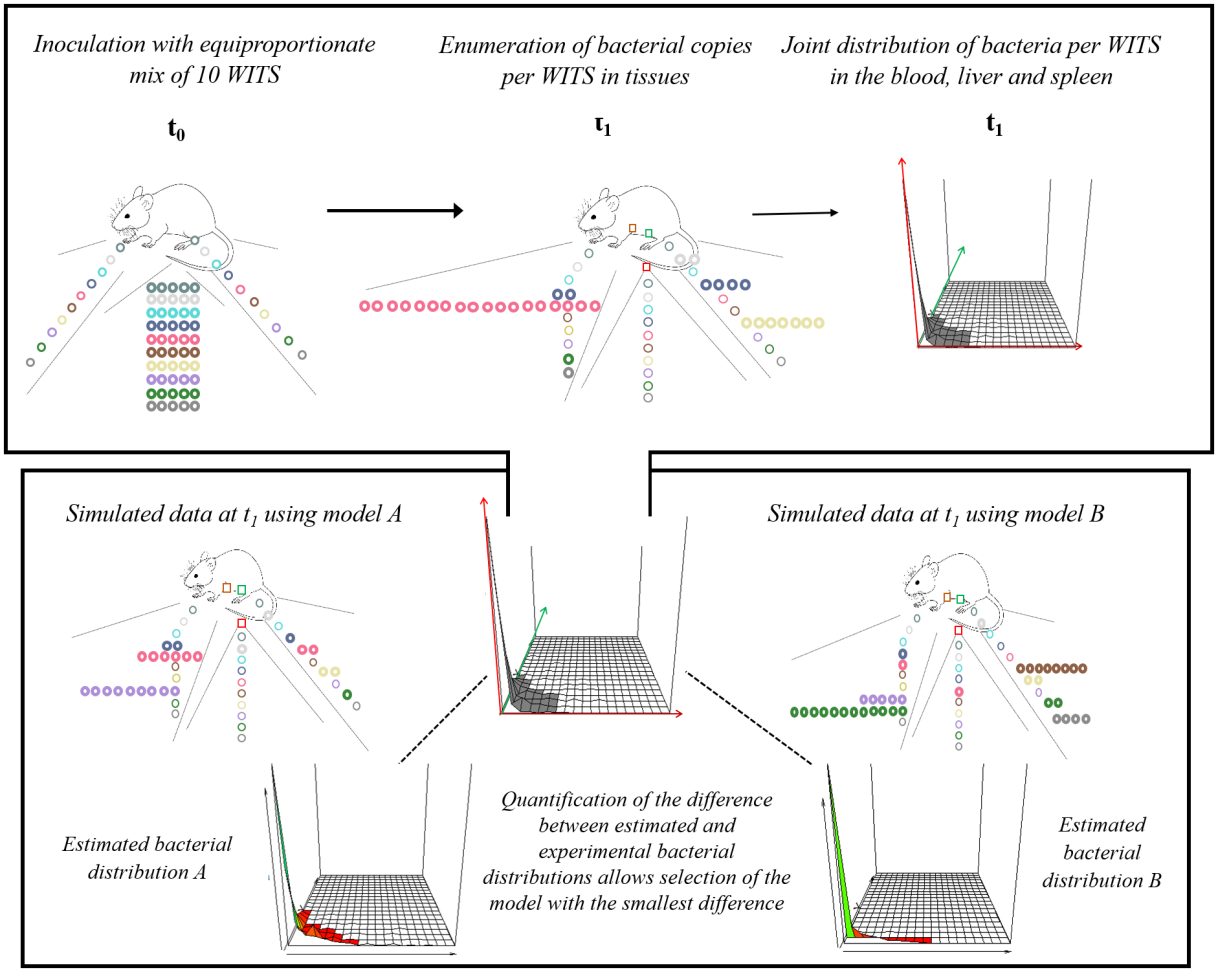
Model selection in a virtual study using tagged strains. Mice are infected with an equiproportionate mix of 10 wild-type isogenic strains (WITS) at t0. At t1, mice are sacrificed and bacterial copies per WITS are enumerated in their blood, liver and spleen. Color-filled circles represent present strains, while unfilled circles represent absent strains. The joint distribution of bacteria per WITS in the 3 tissues uniquely describes the state of the system at t1. Estimated bacterial distributions A and B are obtained at t1 for competing models A and B. Each estimated distribution is compared to the observed distribution and their difference summarized by a divergence measure. Model A yields the smallest difference and, thus, provides a better fit to the data.

However, mathematical models allow for a quantitative assessment of the dynamics of the bacterial infection, as well as model selection. For instance, the observed composition of isogenically tagged populations of bacteria is the result of the convolved effects of replication, killing and migration. By using a within-host model which explicitly represents the processes of replication, killing, and migration, it is possible to precisely estimate these rates by comparing model outputs to experimental data. The precision of parameter estimation is usually reported in the form of confidence intervals (Coward *et al*. 2014; Grant *et al*. 2008; Kaiser *et al*. 2013, 2014; Dybowski *et al*. 2015). Increasing the precision of inference has been achieved by using a larger pool of Isogenically tagged strains (STAMP references) or using inocula with only a small proportion of tagged strains (Kaiser *et al*. 2013, 2014) Apart from quantifying the contribution of different processes in the overall bacterial numbers, it is also possible to assess the plausibility of different biological hypotheses by selecting the model with the best fit to the data (Fig. 3).

Two published studies demonstrate the biological insights offered by complementing the qualitative data interpretation with the quantitative model output. First, Grant *et al*. (2008) demonstrated using the estimates provided by modeling that in the early stages of infection, replication and killing lead to unique subpopulations of bacteria in different infection foci. Estimation of replication and killing rates would not have been possible without using a mathematical model.

Second, Coward *et al*. (2014) introduced elements of mathematical modeling at various points along the experimental timeline. In the early stages of experimental design, simulation-based experiments were used to determine the inoculum dose that would yield the most informative experimental output. Then, mathematical modeling was used to correct the raw experimental data by accounting for the noise introduced due to partial sampling and the samples undergoing qPCR. Finally, mathematical modeling was used to maximize the biological insight by enabling the comparison between the mechanisms of action of the live and killed vaccine. Without models, net growth could be estimated from CFU counts, but distinguishing between the bactericidal and bacteriostatic effects of the two vaccines would have been unfeasible.

Even though mathematical modeling applied on experimental studies using non-phenotypic markers has been widespread in the field of within-host bacterial dynamics, there are limitations. Non-phenotypic marker-based techniques can only capture the dynamics at the bacterial population level, treat the bacterial population in question as having homogenous dynamics and do not provide data in the form of a time series, as different mice are sacrificed at each time point of interest. As a result, the inferences made regarding the rates at which the in-host dynamics evolve can only represent the average of the heterogeneous dynamics at play (Claudi *et al*. 2014). Meanwhile, the assumptions that inter-mice immunological responses are identical and that inter-strain differences remain negligible over time have not been tested. Finally, even in the presence of sufficient data to infer the parameters governing the *in vivo* dynamical processes, mathematical models have hitherto been limited to constant-rate processes, as more complex models become intractable or computationally too expensive to solve.

### Fluorescence dilution-based techniques

Fluorescence dilution techniques take the observation of the within-host bacterial dynamics from the population- to the single-cell level (Claudi *et al*. 2014; Helaine, Cheverton and Watson 2014; Helaine *et al*. 2010; Myhrvold *et al*. 2015). Fluorescence reporter plasmids, in which dilution of a preformed pool of a fluorophore (fluorescence dilution) acts as a measure of bacterial replication, are introduced into a bacterial population. Flow cytometry is used to determine the intensity of the signal and thus the number of replication events that bacteria have undergone.

By virtue of its single-cell resolution, fluorescence dilution techniques are particularly useful to study bacterial heterogeneity. To this day, mathematical modeling has typically not been used for fluorescence-dilution type experiments. Analyzes have focused on qualitatively characterizing the heterogeneity in a signal across a bacterial population, e.g. with regards to its spectrum of replication rates.

**Figure 4.**
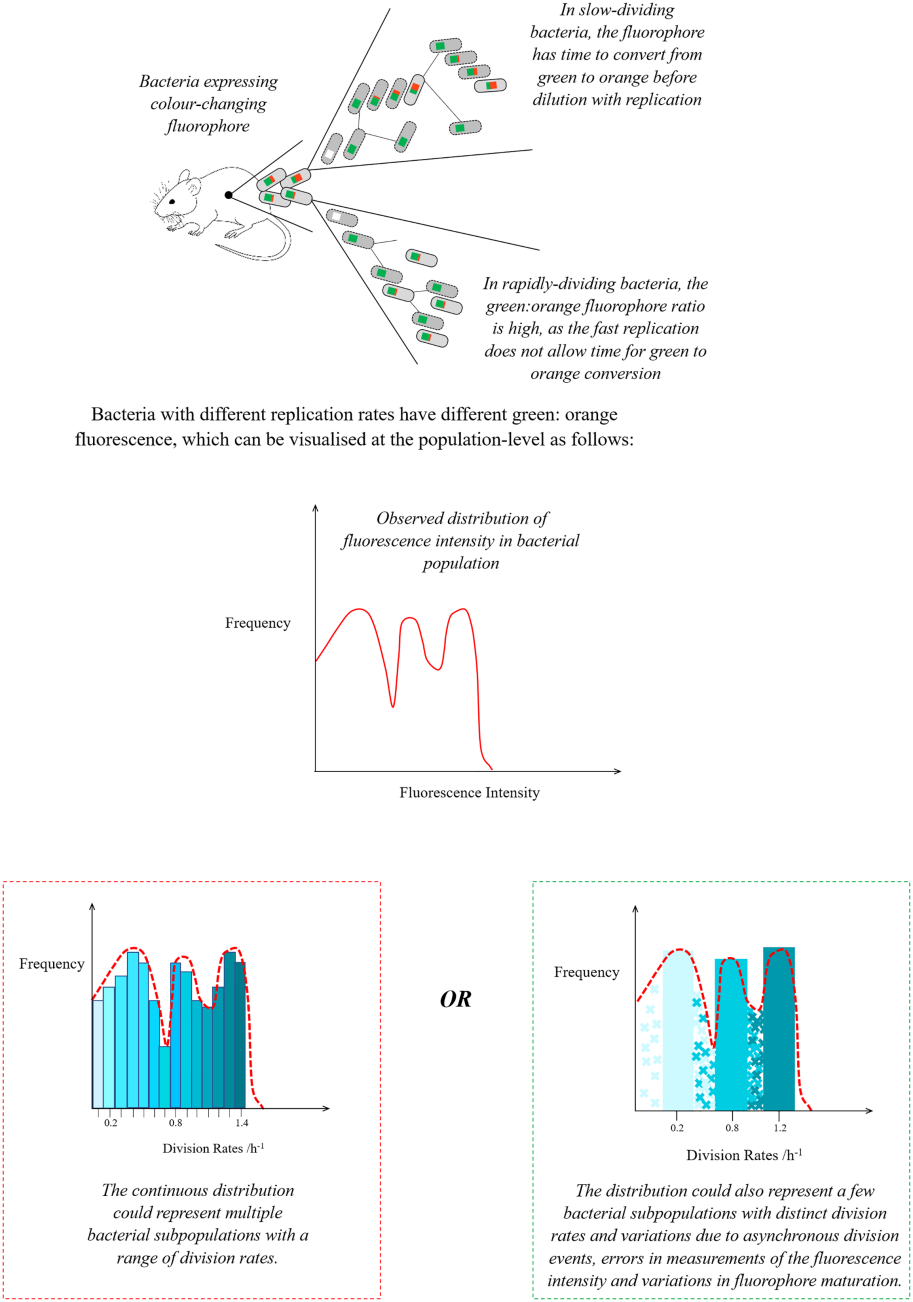
Coupling mathematical modeling with TIMER technique to distinguish between true variation in bacterial division rates and variation due to observational process (adapted from Claudi *et al*. 2014). The observed distribution of fluorescence intensity in the bacterial population appears continuous. Using stochastic models it is possible to quantify the variation expected from different identified sources of noise and compare it to the variation in the experimental data. If the aggregate variation from all sources of noise can account for the variation in the data, it is not necessary to implicate models of higher complexity, such as multiple bacterial subpopulations with distinct division rates.

However, mathematical modeling is needed to precisely quantify the extent of heterogeneity in the biological attribute of interest and to test hypotheses that may underlie this heterogeneity. In theory, because fluorescence halves at each division in fluorescence dilution experiments, discrete peaks should be observed on the histogram of fluorescence corresponding to bacteria at specific numbers of generations. Nonetheless, the histogram of fluorescence intensity is continuous. It is likely that this incongruence reflects some heterogeneity in the experimental process. Mathematical modeling can help distinguish the heterogeneity from the experimental process from the heterogeneity in the biological process. In particular, stochastic models are well-suited for this purpose, because they can capture different sources of variation (Fig. 4).

### Peak-to-trough ratio

One of the most recently developed technique pertinent to facilitating the observation of *in vivo* bacterial dynamics makes use of the differential genetic signatures left by replication cycles (Korem *et al*. 2015). During DNA replication, regions that have already been passed by the replication fork will have two copies while the yet unreplicated regions will have a single copy. The ratio between DNA copy number near the replication origin and that near the terminus, termed peak-to-trough ratio (PTR) should reflect the growth rate of the bacterial population.

In 2018, Gao and Li developed an algorithm allowing the quantitative inference of bacterial growth rates from PTR data, which include DNA segments from various bacterial genomes in a faecal sample. This multi-genomic sample comprises sets of overlapping DNA fragments (contigs), which upon alignment can provide information about the pathogen's identity and replication history. The first step of the algorithm allows for correction of sequencing bias (GC content bias is often reported in next-generation sequencing). This is achieved by using a linear mixed effect model, which corrects the average contig coverage according to the average GC content difference in the set. Quality control and exclusion of contigs is followed by a principal component analysis of contig coverages in multiple samples leading to more accurate inference of the distance between the contig and the replication origin compared to single samples. Quantification of parameter estimate accuracy when using experimental data of variable quality (e.g. different number of contig sizes, degree of contamination, contig number) is possible. This feature could be relevant to experimental design, as it could inform experimental biologists of the trade-off between data quality and level of inference accuracy.

## BIOLOGICAL THEMES EXPLORED WITH THE USE OF MODELS

Sections 2 and 3 have introduced necessary conceptual tools to appreciate the insight that mathematical models can offer when used in conjunction with experimental data. We hope that we have also provided a rough guide of how different formats of data can be explored mathematically. The purpose of the present section is to present biological themes which customarily come up in the study of within-host dynamics of infectious disease and how they have been addressed through the use of mathematical models. As modeling examples remain limited in the area of bacterial dynamics, we include conceptually similar studies in other pathogenic classes including viruses and parasites. These case studies are by no means an exhaustive representation of the entirety of the literature body but have been selected to serve as illustrations of the concepts.

### Quantification of the relative contribution of different immune system components to the progression of infection

Within-host models of parasitic, viral and bacterial infection often seek to determine the relative contributions of different components of the immune system in regulating the dynamics of infection. In the current treatment paradigm, the role of the host's immune response is often neglected, and therapeutic agents are administered for fixed periods of time usually in the form of monotherapy and regardless of the infectious load. It is now becoming increasingly recognized that a first step toward optimization of existing therapies is the induction of synergistic effects between the host immunity and the standalone effect of the therapy (Gjini and Brito 2016). To thoroughly understand this interaction, mechanistic mathematical models can be used in two main ways. First, one can use a series of nested models prospectively, starting from simpler models and adding features of the immune response while quantifying the impact of each new addition in the process. Second, by enabling the segregation between unobserved processes of replication and killing, mathematical models begin to shed light on the black-box of host–pathogen interactions and inform further biological experiments. For example, if rapid killing is identified as the main driver of an observed decline in bacterial numbers, it is reasonable to first look in the direction of known cidal branches of the host immunity.

One such approach was taken by Grant *et al*. in 2008 where following the identification of early bactericidal activity in a *Salmonella* mouse model, infection progression in wild type mice was compared to that in NADPH oxidase deficient mice to unravel an important role of that immunological component in inducing the inferred bactericidal effect. In other pathogen classes, mathematical models addressing similar questions were successfully used much earlier.

With regards to host immune system-bacterial interactions, mathematical modeling of the *Mycoplasma* species has been ongoing. The Kirschner group have developed a series of increasingly complex mathematical models to describe the role of different cell types and chemokines of the immune system in the progression of early tuberculosis (TB) infection. Their compartmental model based on ordinary differential equations including the lung and the draining lymph node (DNL) has been used to study the dynamics of early infection, particularly the role of dendritic cells in T-cell priming (Marino and Kirschner 2004) and, later, the roles of dendritic cell trafficking to and from the DNL (Marino *et al*. 2004), cytotoxic T-cell-mediated *Mycobacteria* killing (Sud *et al*. 2006), TNF-α and anti-inflammatory IL-10 (Cilfone *et al*. 2013) in host defence. Their contributions identified the macrophage infection rate and T-cell-mediated immunity as the two key elements in determining the trajectory of an infection into one of (i) primary TB, (ii) primary TB with clearance, (iii) latency and (iv) reactivation (Marino and Kirschner 2004).

### Comparison of the effect of different strains on infection dynamics

Fitting within-host models to samples of different strains of the infectious pathogen can also facilitate our understanding of how the within-host dynamics of infection vary across different strains of the same species. Different strains of pathogens are responsible for differences in seasonal and local outbreaks of contagious and deadly infections such as influenza (Du *et al*. 2017), cholera (Weill *et al*. 2019), community-acquired pneumonia (Zhang *et al*. 2019) and others. These pathogens, albeit very closely related, can show extreme differences in transmission rates (e.g. in *Mycobacterium tuberculosis* in Verma *et al*. 2019), response to therapeutic agents (e.g. in *Vibrio cholerae* in Weill *et al*. 2019) and virulence (e.g. in swine fever virus in Portugal *et al*. 2014). In this context, mathematical models allow for sensitivity analyzes to identify which parameter(s) have the greatest impact on a given outcome; this can be helpful in highlighting potential causes that drive inter-strain differences.

For instance, Hur *et al*. (2013) fit models of influenza infection to experimental data on seasonal and pandemic strains of flu. They found that the only parameter that varied between the pandemic and seasonal strains was the viral replication rate, indicating that intracellular viral replication may affect pathogenicity.

### Comparison of the effects of different therapeutic interventions on infection dynamics

Within-host models of infection (both theoretical data-driven) can also reveal important insights into the effect of different drugs at the level of the host–pathogen interaction and identify effective treatment strategies (e.g. decide whether it is more efficient to prevent replication or increase killing). For example, Rong and Perelson (2010) evaluated the effect of different Hepatitis C (HCV) treatment strategies. Protease inhibitors are being increasingly used in combination with pegylated interferon and ribavirin to treat HCV-1 infection, but there remain concerns of relapse after treatment. They developed a deterministic mathematical model to examine viral load dynamics before and after treatment with a protease inhibitor. Banerjee, Keval and Gakkhar (2013) considered the effect of ribavirin being used in combination with interferon therapy for HCV infection. Although the study was theoretical in nature, it found that – provided a certain threshold of drug efficacy – a triphasic response of viral load could be observed, leading to eradication of the virus.

### Comparison of the effects of different inoculum size on infection dynamics

Infections can take off with inocula of variable sizes. However, the inoculum size affects the population composition of the infectious agents and how they respond to therapy. Formulating a deterministic mechanistic model, Meredith *et al*. (2015) reported that inoculum size determines the efficacy of *β*-lactam antibiotics when administered to bacterial populations of which at least some members harbor extended *β*-lactamase activity. If *β*-lactam antibiotics were administered in high-density populations, then some members would survive and re-establish the infection. They reported that the population was sensitive when its initial density was sufficiently low or examined in a short time window. Given these properties, they reasoned that optimal antibiotic dosing may remain effective in bacterial populations even when they harbor resistance genes.

### Studying the dynamics of infection across different scales

Mathematical models can be employed to study host–pathogen interactions at multiple levels, from cellular to whole-organism and even population level. A solid knowledge of the versatility of mathematical techniques allows the use of the same tools to study questions on different scales. With judicious use, mathematical models can also combine insight acquired at different levels e.g. the single cell and organ levels and use this to gain novel insights about disease progression (Gog *et al*. 2015). For example, a stochastic mathematical model generated by the Perelson lab showed that early HIV dynamics differ depending on whether infected target cells produce virions continuously or do so in a single burst (Pearson, Krapivsky and Perelson 2011). This study shows how events at the single-cell level can have a profound impact on infection dynamics at the whole-organism level ultimately affecting clinically important quantities used for diagnosis and as guides for therapeutic intervention.

Furthermore, it is possible to use the predictions from modeling the host–pathogen interactions to inform models at higher scales. In 2009, Heffernan and Keeling (2009) took advantage of well-founded predictions about immunity in a measles-infected host (Heffernan and Keeling 2008) to predict the effect of vaccination at the population level.

### Investigating the evolutionary dynamics of infectious disease within the host

Finally, mathematical models have been used to characterize and quantify the evolutionary dynamics of infectious agents within a host. For instance, Chisholm and Tanaka (2016) developed a mechanistic mathematical model to examine the evolution of *M. tuberculosis* within its host. *M. tuberculosis* is observed to enter a latent, dormant state, but, at first glance, a state of dormancy is not advantageous for the pathogen as it does not permit replication. However, the study demonstrated that latency can be an evolutionarily desirable state.

Furthermore, Fabre *et al*. (2012) formulated a deterministic mechanistic model of competing viral populations within host plants. They parameterized it according to the carrying capacity of the plant, the intrinsic rate of increase of each variant and the competition strength each genotype exerts on the others. They determined the forms of selection processes occurring between competing viruses within a host plant, and the intensity and temporal variation of genetic drift experienced by viruses during host plant colonization. Parameters were statistically inferred by model fitting to high-throughput sequence data of the viral counts obtained from the plants over time, and model selection was performed (after testing several models reflecting different mechanisms of competition).

## CONCLUSION

This review attempted to bridge the gap between our increasing observational capacity in the lab on one hand, and the mathematical modeling approaches whose computational efficiency is improving on the other. Experimental techniques aiming to observe bacterial replication, killing and migration are capable of higher and higher resolution. They yield rich experimental outputs, which *per se* suffice to gain meaningful qualitative insights on the dynamics of infection. Nevertheless, sole qualitative assessment of the experimental output constitutes a significant underuse of resources. When in conjunction with bespoke mathematical models, the same raw data can be used to segregate between unobserved processes when solely their convolved effects are observed, to quantify the rates at which these processes occur, and to test competing hypotheses regarding the underlying biological mechanisms. Ultimately, a quantitative measure of the likelihood of a biological hypothesis can inform resource allocation in further experimental studies.

Furthermore, the role of mathematical modeling in optimizing experimental conditions should not be overlooked, as an experimental protocol design that ignores the modeling aspect is set up to obtain data that would most likely be suboptimal for modeling (Succurro, Moejes and Ebenhöh 2017). As a result, it is crucial that modelers and experimentalists come together at the conceptual stages of a project to jointly plan experiments, measurement frequency and time-points and data management.

Due to limited cross-disciplinary training, this dialogue has not been extensive so far. Nevertheless, attempts to pair certain forms of experimental output to certain mathematical modeling techniques have begun to emerge recently, as shown conceptually in Section 2. This creates a standardized platform that makes modeling more accessible to those with less expertise and highlights the recognition of the added value of bringing mathematical models and experimental data together.

In recent years, decisive steps have been taken in the direction of establishing customizable modeling platforms corresponding to specific data formats. First, Price *et al*. (2017) developed a freely available package in the R-programming language facilitating the implementation of mathematical models on DNA barcoding data. Second, Gao and Li (2018) developed an algorithm allowing the quantification of bacterial replication rates from PTR data. In recognition of the added value that mathematical models can provide when combined with experimental data, these efforts highlight the recently increasing interest in expanding the dialogue between experimentalists and modelers using tools that can be understood and used by both parties. We hope that by presenting a pairwise overview between experimental techniques and mathematical modeling approaches, we have not only illustrated the versatility of models in addressing a wide range of biological questions but also provided the impetus for microbiologists to reconsider the role of modeling at all stages of the experimental procedure.
